# Field deployable impedance-based corrosion sensor

**DOI:** 10.1038/s41598-021-03840-5

**Published:** 2022-01-07

**Authors:** Jiajun Li, Xiaoxue Jiang, Faheem Khan, Xuanjie Ye, Shuren Wang, Jie Chen

**Affiliations:** 1 Department of Electrical and Computer Engineering, Electrical and Computer Engineering Research Facility, University of Alberta, Edmonton, AB T6G 2V4 Canada; 2 Fourien Inc., 4407, 68 Ave NW, Edmonton, AB T6B 2N2 Canada

**Keywords:** Electrical and electronic engineering, Chemistry

## Abstract

Electrochemical impedance spectroscopy (EIS) has been used in various applications, such as metal corrosion monitoring. However, many conventional corrosion monitoring setups are bulky and inconvenient for in-situ testing. The purpose of this work is to reduce the size of the whole corrosion monitoring system. We utilized EIS to design a field deployable impedance-based corrosion sensor (FDICS), capable of performing in-situ EIS analysis. Experiments verified the sensor’s accuracy, and the results showed that the sensor performed similarly to a bench-top EIS machine when we tested on circuit models. Furthermore, we used the proposed FDICS to monitor a metal corrosion experiment and performed EIS. The result showed that the proposed FDICS is able to obtain the sample’s impedance spectroscopy, which could help researchers test its corrosion severity on a metallic sample in-situ. Compared to other bulky conventional setups, our device eliminates the design complexity while still showing insights into the corrosion reaction.

## Introduction

Due to their excellent electrical conductivity, thermal conductivity, and ductility, metallic materials, such as steel, have been widely used in making oil pipes^1^. However, one of the disadvantages of typical metallic materials is that they are easily corroded, especially when exposed to an electrolyte solution, resulting in electrochemical corrosion on the surface^2^. Once the metal’s structural strength is destroyed by corrosion, oil leakage occurs, which often results in unnecessary economic losses and environmental pollution^3^. In order to track corrosion, we need to monitor the corrosion reaction in real-time so that proper and timely interventions can be taken in advance.

Electrochemical Impedance Spectroscopy (EIS) analysis is a very effective way to monitor metal corrosion. One of the advantages of conducting EIS analysis is that we can better understand reactions on the electrode with relatively simple steps and analysis^4^.In the EIS analysis, the magnitude and phase of the sample’s impedance at different frequencies are accurately measured. There are two main modes for monitoring the electrochemical reactions through EIS, i.e., the galvanostatic mode and the potentiostatic mode. Galvanostatic EIS analysis is mainly used in the development and research of large power devices such as batteries, where a current is fed into the sample, and the voltage signal was measured^5,6^. For monitoring metal corrosion, potentiostatic EIS is the most commonly used method^7^. At different frequencies in potentiostatic EIS, the required DC voltage across the sample is relatively stable. A small AC disturbance voltage signal is applied across the sample under test. Once the AC current through it and the AC voltage across it are known, the sample’s complex impedance can be calculated^7–9^. Also, EIS can be seen as a way to find the transfer function of the electrochemical system by comparing the input and output signals since the sample-under-test is regarded as a “Black-Box”^10^.

EIS has been used in corrosion analysis for decades. Its non-invasive nature and easy operation made it easy to perform quick and preliminary analysis. For comprehensive metal corrosion analysis, a single EIS analysis is not enough. Other techniques such as scanning electron microscope(SEM) should be used to find out the structure change of the sample-under-test^11^. Many research groups have studied the corrosion of metal materials.EIS techniques have been used to investigate metal corrosion inhibition^9^. However, a bulky EIS instrument is generally required^12,13^. Although large instruments tend to provide more accurate EIS data, it is very inconvenient to carry large such bulky instruments for on-site measurements. The on-site environmental uncertainty could also damage the instrument. Therefore, small and portable instruments for potentiostatic EIS analysis are desirable.

A lot of research has been carried out to miniaturize the EIS device. Yu et al. developed an impedance detection circuit with a 3% error comparing to an LCR meter^14^. The device is designed to detect different bio-molecules on a portable biosensor system. Jiang et al. have developed an EIS system that can be used as a bio-detection system^15^. The weight of the device is only 450g, and the cost is about $45. The performance of the device is as follows. The frequency sweep is from 100 Hz to 500 kHz, the measurement range is 10 Ohm-100 kOhm, and the phase accuracy reaches 0.936. In their experiment, the instrument carried out EIS analysis on eggs at different times during the heating process. Ferreira et al. designed a bio-impedance monitor system with a 4-electrode analog front-end (AFE) for wearable plethysmography applications. The working frequency is between 0 and 100 kHz, and the error is less than 1%^16^. The working frequency range of the design is 10 Hz–0 MHz. In the actual measurements, magnitude data can be obtained within the frequency range of 10 Hz–3 MHz. Relatively accurate phase measurements can be obtained at 10 Hz–20 kHz. Rajabzadeh et al. developed a fast multi-electrode arrays (MEA) EIS system with 24 channels. The system is able to detect an impedance range from 6 $$\Omega$$ to 300 k$$\Omega$$ in the frequency range of 10 mHz to 100 kHz. The system was used for in-vitro biological studies and showed high accuracy comparing to the commercially available equipment^17^. Sebar et al. developed an EIS system based on a teensy board. The system is able to operate from the frequency range from 0.01 to 50,000 Hz. For accuracy, the system can measure the impedance of 50 k$$\Omega$$ while maintaining an error within 5%. The system is dedicated to the electrochemical cells with three electrodes^18^. Besides the impedance measurement systems reported in the literature, many commercialized devices can perform EIS analysis in a wide range of applications. Other than the EIS analyzer available on the market, such as the devices from PALMSENS and AUTOLAB, the proposed FDISC specially designs for metal corrosion analysis, and the system complexity is greatly reduced. Meanwhile, the cost of commercialized devices generally starts from 10,000 USD, while the proposed FDICS costs hundred of dollars.

In order to minimize metal corrosion monitoring system, Grassini et al. designed a miniaturized EIS system using Arduino, and the measured frequency range is 0.01 Hz–100 kHz. It is worth mentioning that the stimulus amplitude ranges from 10 mV to 2 V. This instrument performs EIS analysis on cultural relics exposed to the outdoors, and the main component of relics is metal. Because the main components of the entire system are two Arduino development boards, the entire system is very light and portable^19^.The conventional corrosion acceleration system keeps the solution static while rotating the metal sample during the test, resulting in bulky motors, thus increasing the upfront price and long-term maintenance. Circulating the solution rather than rotating the metal sample eliminates the need for a large setup and can be performed in a miniature flow cell.

## Method

For this study, our method to achieve metal corrosion is to design the hardware and software of the proposed FDICS. In this section, we illustrate the design of the proposed FDICS. Each part of the design will be introduced.

**Figure 1:**
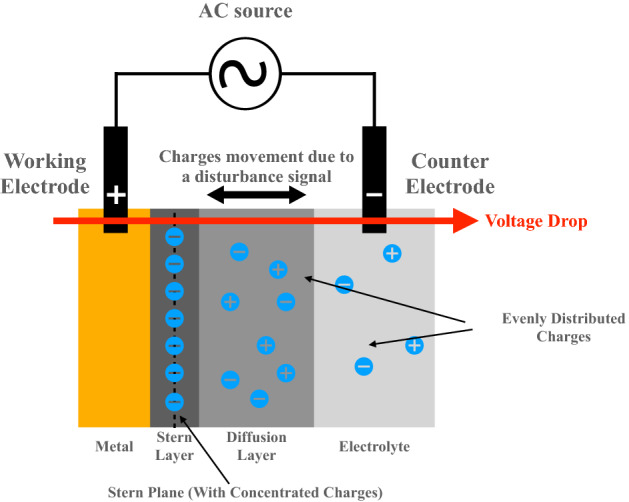
Charging and discharging of the double layer caused by the AC signal.

When a metallic sample immerses in the electrolyte solution, reactions occur between the solution and the metal. Because of the reaction, there will be charges accumulate on the surface of the metal. For these accumulated charges, we can divide them into different layers. They are the Stern layer, and the diffuse layer (or the double layer)^20,21^. In the EIS analysis, a small disturbance sine signal (input) is applied on the double layer from one end of the sample, forcing the charges to move towards or away from the metal. At another end, the response signal (output) is collected. The input and output signals are slightly different from each other due to the energy loss when the signal passes through the sample. Comparing the input and the output sine waves, we can find information on the corrosion process. Specifically, we can find the complex impedance of the sample^7^. Figure 1 shows the charging and discharging of the double layer caused by the AC signal. In order to measure the sample’s impedance, a two-electrode potentiostatic device is applied. A little disturbance signal is given to the sample, which results in the charging and discharging effects of the corroding metal double layer. In the EIS analysis for metal corrosion study, the working electrode is attached to the metal sample, and it is where the potential is maintained. The counter electrode immerses in the solution, and the electrochemical cell is connected to the EIS system.

**Figure 2:**
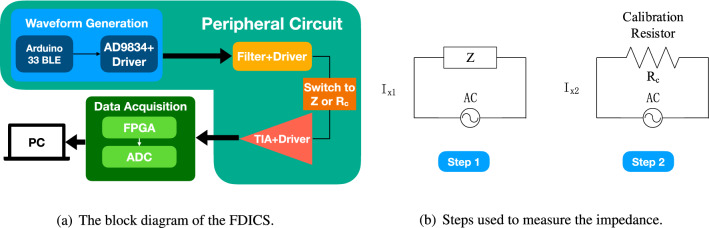
The block diagram of the proposed FDICS and the steps to measuring the impedance.

The portable potentiostatic device was implemented using both the hardware and software. As shown in Fig. 2a, the hardware design of our device consists of several modules. They are peripheral circuits, field-programmable gate array (FPGA)-controlled analog-digital converter (ADC) to convert the signal from analog to digital, and a personal computer (PC). The entire workflow is as follows. First, the Arduino sends control signals to AD9834 to generate a series of sine signals, which are applied to the peripheral circuit. The signals pass through the filters and drives, and then signals were applied to the sample impedance (*Z*) or calibration resistor ($R_{C}$). The ADC then converts the voltage at the output of the trans-impedance amplifier (TIA) to a digital signal. The FPGA sends the signals to the PC for signal processing and impedance calculation.

### Peripheral circuit

In general, the purpose of the peripheral circuit in the proposed FDICS is as follows:

1. Generate a sine wave and level down the amplitude of the signal (to about 50 mV) to make sure the proposed FDICS is a potentiostat^7^.
2. Filter out noises that could cause inaccuracy on the impedance measurement result.
3. Switching the signal between *Z* and $R_{C}$.
4. Transform the current signal that yields during testing to a voltage signal. And then amplify the voltage signal to a proper amplitude for ADC sampling.

An AD9834 20 mV 75 MHz DDS (Digital Direct Synthesis) controlled by an Arduino 33 BLE functioned as the signal generator of the whole system. A series of sinusoidal signals were generated by AD9834. There are four groups of signals in total, as shown in Table 1, each corresponds to a specific ADC sampling frequency. The AD9834 has a frequency resolution of 0.28 Hz under a 75 MHz oscillator. The selection of 10Hz as the starting point of the testing signal is a trade-off between the testing speed and accuracy. The selection of sampling frequencies is based on the signal frequency. According to the Nyquist law, if the sampling frequency is lower than twice the signal frequency, aliasing occurs. Therefore, as a requirement of the measurement, there is a minimum value of the sampling frequency. Table 1 shows that from 10 Hz to 200 kHz, the sampling frequency is at least 2.5 times higher than the highest signal frequency in the corresponding group. 100 MHz is the sampling frequency for the last group, which ideally could handle frequencies up to 50 MHz. However, due to the parasitic capacitance and inductance in the system, we could only obtain certain results under 1MHz. For the last group, we used a higher speed ADC than the other three groups. The purpose is to perform faster sampling for the signal. In the first three groups, we used a 12-bit ADC with the highest sampling rate of 500 ksps (kilo samples per second). The ADC used in the last group is 14-bit, and its highest sampling rate is 150 Msps (million samples per second) sampling rate high-speed ADC. These two different ADCs are on the commercial board P0435 (Terasic Technologies, Hsinchu, Taiwan). In Table 1, the sampling frequency is converted through the sampling rate.

**Table 1:** The signal frequency and the corresponding ADC sampling frequency.

Signal Frequency	10–400 Hz	500 Hz–40 kHz	50 kHz–200 kHz	300 kHz–1 MHz
Sampling Frequency	10 kHz	100 kHz	500 kHz	100 MHz

Before the signals are applied directly to the corrosion sample, a 6th-order Butterworth low-pass filter is used to minimize the high-frequency noise that may influence the measurement result. Butterworth filter is a very commonly used filter. We selected the 6th order because the result is a trade-off between the design complexity and the performance. We use a low-pass filter to eliminate high-frequency noise^15^. For example, the frequency of the quantization noise from the digital-analog converter (DAC) in the AD9834 DDS is higher than the signal frequency. The filter circuit removes undesired noise to obtain more accurate impedance measurement results. Driver circuits are also used before the input and after the output of the filter. The driving circuits are simply inverting op-amp circuits^22^. Generally speaking, for blocks with input and output signals, we intend to increase the input resistance and decrease the output resistance to isolate different stages of the system. Also, the driving circuit at the front of the low-pass filter leveled down the signal amplitude. Proper signal amplitude is required in EIS analysis on the metal corrosion phenomenon^8^. Meanwhile, the driving circuit can guarantee that the signal output is relatively stable so that the proposed FDICS can function as a potentiostat.

An analog switch 74AUP2G57GUX (Nexperia BV, Nijmegen, Netherlands) controlled by the Arduino in the circuit can switch the signal between the calibration resistor and the corrosion sample. The switch is very suitable for FDICS due to its low-power consumption, wide supply voltage range, high-frequency, and high-noise immunity. Figure 2b shows how we calculate the sample impedance (*Z*) using a calibration resistor ($R_{c}$). The first step is to connect the test sample to be tested and measure the current $\hbox {I}_{{x1}}$. The second step is to apply a voltage to the calibration resistor to get the current $\hbox {I}_{{x2}}$. Impedance can be obtained by Eq. (1). The $\theta _{1}$ and $\theta _{2}$ are the phase of the impedance of the sample and the calibration resistor respectively. For the calibration resistor, the phase = 0. As mentioned above, the switching between *Z* and $R_{c}$ is obtained using an analog switch.

1 $$Z=\frac{R_{c}\cdot I_{x2}*exp(j\omega t-\theta _{2})}{I_{x1}*exp(j\omega t-\theta _{1})}$$

The output from the sample or calibration resistor is a current signal. However, a voltage signal is required for ADC to sample, and thus a trans-impedance amplifier (TIA) is required. The gain is about 35. Under this condition, amplifying a signal of several tens of millivolts could yield an output signal with a peak-to-peak value below 3.5 V. The ADC input range is 0–4.096 V, which is high enough for a signal with a 3.5 V peak-to-peak value so that the ADC can function properly. This is the reason why we choose the gain of 35.

Overall, the peripheral circuit’s function is to generate a sine disturbance signal and feed it to a corrosion sample or a calibration resistor. Finally, by increasing the signal amplitude, the ADC can sample the signal more accurately.

### ADC sampling and data acquisition

Using the ADC, we converted the obtained analog signals into digital signals and processed the digital signals using a commercial board P0435 (Terasic Technologies, Hsinchu, Taiwan). On the P0435 board, there is an FPGA that can control the onboard ADC. We also select this board because of its portability and simplicity of re-programming. The operating speed of this board is fast enough to perform the data acquisition. The signals were then sent to the PC for data processing. In this process, the synchronization between signal generation and data acquisition is crucial. Since the Arduino and FPGA are two different blocks, we set a 2-s time delay for each testing frequency in the Arduino code and FPGA code separately. The purpose of the 2-s delay is to want the system to settle down from the previous disturbance signal. We found that 2 s was enough to perform the impedance measurement under the next frequency in our testing. For the EIS analysis, the system has to be stable, casual, and linear^7^. The 2-s delay is properly set and guaranteed that the system can get rid of the previous distribution signal so that the system in the corrosion cell can be stable. At a specific time interval, the sine signal’s output frequency from the AD9834 corresponds to the ADC sampling frequency. As a result, we can obtain the impedance result at a particular frequency.

Table 1 shows four different sampling frequencies that correspond to each group of sine testing signals. The ADC is controlled by a processor generated through Nios II App. The digital signal is transmitted to the PC, and the data was saved into four different files according to the different sampling frequencies. After storing all the data into different files, we used them one by one for impedance calculation. For each separate file, the data was read to a cell array first. We then calculated the frequency, magnitude, and phase of the signal. By performing a Fast Fourier transform (FFT), we can obtain the magnitude and phase of the signal as shown in Eqs. (2) and (3). In Eq. (2), $\hbox {I}_{{x2}}$ [k] and $\hbox {I}_{{x1}}$ [k] are the FFT magnitude result of the current through the calibration resistor and the sample under test, respectively. For the phase in Eq. (3), $\theta _{x2}$ and $\theta _{x1}$ are the FFT phase results of the current through the calibration resistor and the sample under test, respectively.

2 $$Amplitude=\frac{I_{x2}[k]*R_{CAL}}{I_{x1}[k]}$$

3 $$Phase=\theta _{x2}-\theta _{x1}$$

**Figure 3:**
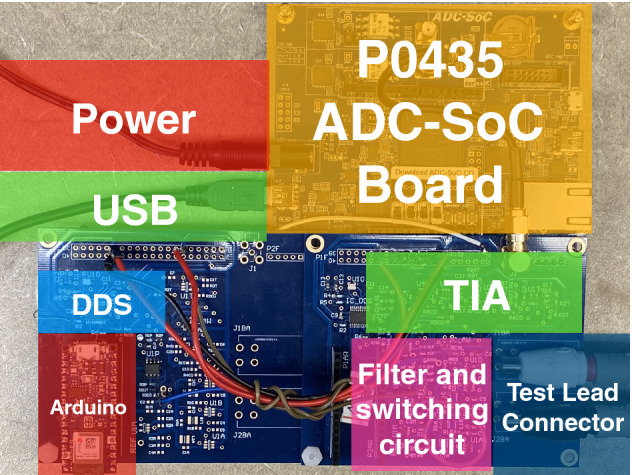
The proposed FDICS.

Figure 3 shows the implemented FDICS. Some areas were colored with explanation, showing the position of different blocks.

## Results

The method of instrument evaluation used in^15^ for instrument evaluation can be used to analyze our experimental results. The entire instrument evaluation process in this paper is divided into two stages: the first stage is precision verification and the second stage is EIS analysis.

- In the first stage (precision verification), we used our instrument to measure resistors. The resistance is known in advance. We then calculate the accuracy rate based on the measured values. Secondly, we measured a test sample consisting of two resistors and a capacitor. Finally, we compared the measurement results between the proposed FDICS and the bench-top SP-200 EIS instrument.
- In the second stage (EIS analysis), we measured the EIS impedance spectra of a corroded metal sample in a 10-h experiment at different times and then used the equivalent circuit for parameter fitting. The fitting result can be explained by knowledge in the reported literature.

### Precision verification

For the precision verification, a series of resistors ranging from 10–150 $\Omega$ were used as the testing samples. These resistance values were selected because the impedance of the corrosion cell is within the range. The operating frequency used is 10 Hz–200 kHz. The resistor used in the experiment is the RS-201 resistance substituter (IET LABS INC. Long Island, New York, USA) with an accuracy of (0.1% + 0.025 $\Omega$). Since we are going to measure the magnitude and the phase of the resistors, we will calculate the magnitude and the phase error separately. Comparing with the FDICS measurement results to the values of the pre-set resistance substituter, the measurement magnitude result error was calculated using Eq. (4) for each of the values of the resistors under a certain signal frequency. As a result, we can get a series of calculated errors for each resistor under a certain frequency. Taking the average of all the error data we collected, we can get the magnitude error = 0.028 with standard deviation = 0.02489.

4 $$error=\frac{|Z_{measurement}-Z|}{Z}$$

The ideal phase of the complex impedance of a pure resistor is 0, so we regard the absolute value of the phase result as the phase error. Table 2 shows the results of the error calculations. The phase error of the device is calculated by taking the average of all the calculated error results. In this case, for the proposed FDICS, the phase error is 1.051 degrees with standard deviation = 1.21699. The inaccuracy could come from the limitation of the device, such as the resolution of the DDS and ADC. Also, the parasitic capacitance in the whole system contributes to the inaccuracy in the final measurement result.

**Table 2:** The impedance magnitude and phase measurement error.

Frequency (Hz)$\backslash$resistance ($\Omega$)	10	20	50	100	150	10	20	50	100	150
10	0.088	0.043	0.002	0.001	0.011	0.448	0.297	0.102	0.200	0.233
100	0.064	0.022	0.008	0.016	0.024	0.020	0.017	0.064	0.160	0.076
1000	0.063	0.022	0.008	0.017	0.022	0.367	0.069	0.060	0.105	0.047
10k	0.063	0.022	0.008	0.018	0.020	0.001	2.980	2.101	1.496	1.311
100k	0.072	0.025	0.007	0.017	0.021	2.813	0.139	3.256	3.447	2.657
200k	0.089	0.030	0.006	0.018	0.021	1.104	3.073	0.590	2.669	1.621
	Magnitude result error					Phase result error				

**Figure 4:**
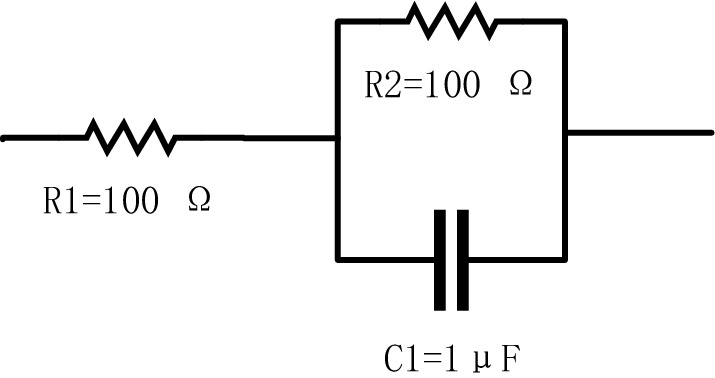
The circuit model as sample under test.

The next step is to compare the proposed FDICS and a bench-top commercial instrument, the SP-200 Potentiostat (Biologic, France). This impedance meter is a single-channel transportable potentiostat workstation. To compare the results, a sample is required. Figure 4 shows the circuit model that was used as the sample for testing. The circuit model can represent the electrochemical behavior of a homogenous metal/solution interface^23^.

**Figure 5:**
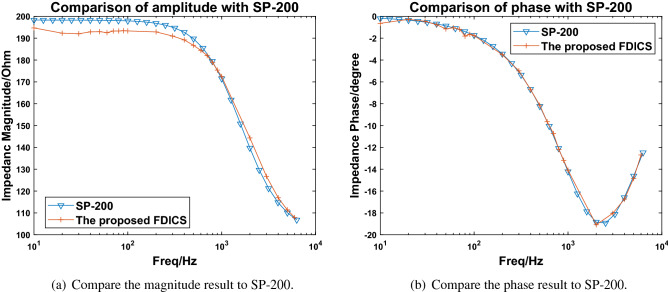
The comparison between the results from the FDICS and the SP-200 when testing on the circuit model.

The selection of parameters is based on the equivalent circuit parameter fitting result from the EIS spectra of a corrosion cell. Figure 5a, b show the comparison of the measurement magnitudes and phases, where the starting frequency is 10 Hz, and the stop frequency is 6kHz. The results of the proposed FDICS showed the same trends as the commercial SP-200. Our results are slightly lower than those of the SP-200 within the whole testing frequency range. The inaccuracy of the measurement result is likely due to the limited accuracy of the AD9834 DDS. At low frequencies, the result deviates from that of the SP-200. Replacing the crystal oscillator (75 MHz) to a smaller value would improve the result. However, in that case, the frequency range would be shorter after replacing the crystal oscillator. The impedance magnitude of the sample decreases over frequencies because the circuit contains a capacitor and two resistors. Equation (5) shows the impedance of the circuit in Fig. 4. R2 is connected in parallel with C1, and the whole is connected in series with R1. $\omega$ is the angular frequency of the signal passing through it.

5 $$Z(\omega )=R_{1}+\frac{R_{2}}{1+j\omega R_{2}C_{1}}$$

Because of only one capacitor in Fig. 4, there is only one time constant in the circuit. As the frequency increases, the magnitude gradually drops. At low frequencies, the results about equal to $\hbox {R}_{{1}}$+$\hbox {R}_{{2}}$. At 6 kHz, the results approximately equal to the value of R1. Up to 6 kHz, the performance of FDICS is good. The conclusion of the device’s performance is capable of performing in-situ EIS analysis is drawn based on the testing result on the circuit model when the frequency is up to 6 kHz and the comparison between our work and other works such as^14,19^.For the phase results, the two systems matched perfectly. In all, FDICS showed good compatibility and accuracy, and thus we can use it for the EIS analysis for the corrosion study.

### EIS analysis with a corroded metal sample

**Figure 6:**
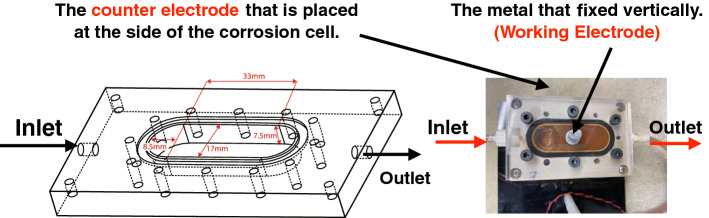
A 3-D diagram showing the dimensions of a corrosion cell (Left). A real corrosion cell (Right).

Corrosion cells are often used to perform EIS analysis^8^. Consequently, a simple and portable corrosion cell was prepared for the in situ experiments^24,25^. Figure 6 shows the corrosion cell that we used in our experiments. The corrosion cell (model Deptrak D100) was manufactured by Fourien Inc. (Edmonton, AB, Canada). During a corrosion experiment, a metal sample is fixed vertically on the corrosion cell. One end is exposed to the air, and the other end is exposed to the solution. The purpose of positioning the metal sample in such a way is to build a direct connection between the corrosion cell and the measurement system to minimizing any errors. There are inlet and outlet tubes connecting the two ends of the corrosion cell. By circulating the electrolyte solution (3.5% NaCl, about 2 ml/s speed) in the corrosion cell rather than rotating the metal sample in the solution as a conventional corrosion cell, we get rid of a bulky motor and reduce the cost of the metal corrosion monitoring setup. Also, the chamber can contain gases inside to participate in the corrosion reaction, which is desired in some corrosion studies^26^. 3.5% NaCl solution is commonly used in the metal corrosion experiment; for our experiment, we adopt this 3.5% NaCl solution^8^. During experiments, a peristaltic pump drives the solution to flow cyclically from the inlet to the outlet. For different experiments, we can easily replace metal samples. Table 3 shows the composition of the metal sample that we use for the corrosion. The sample is categorized as low-carbon steel (LCS).

**Table 3:** The composition of the low carbon steel sample.

Material	Composition
Carbon	0.13–0.20%
Manganese	0.30–0.90%
Phosphorus	0.04% Max
Silicon	0.15–0.30% Max
Sulfur	0.50% Max
Iron	Balance

**Figure 7:**
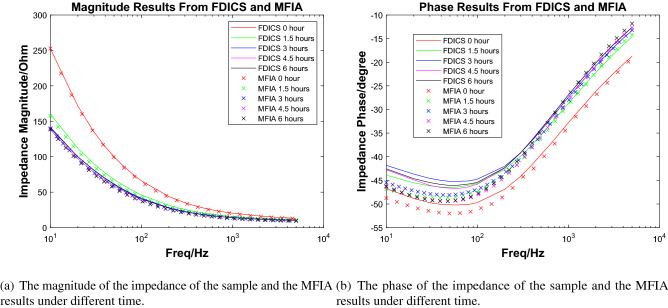
The comparison between the results from the FDICS and the MFIA when testing on the corrosion cell.

In the experiment, the peristaltic pump’s 3.5% NaCl solution was driven to flow through the metal sample in the cavity evenly. At the beginning of the experiment, the liquid in the cavity was clear and transparent. However, after 6 h, some precipitations began to appear in the cavity because the iron in the metal sample was oxidized and became rust. Figure 6(right) shows the color of the liquid in the cavity when precipitation occurred. In a 72-h experiment from^8^ of a single-layer coating system in the 3.5% NaCl solution environment, the impedance changed significantly in the first 4 h. For our experiment, a time period of 6 h is enough to observe our sample’s corrosion phenomenon. We measured the impedance of the sample every 1.5 h and recorded the results. In order to compare the FDICS with the bench-top impedance analyzer, throughout the experiment, we collected the impedance data with the FDICS and the MFIA 500 kHz/5 MHz Impedance Analyzer (Zurich Instrument, Zurich, Switzerland). Five groups of data were collected with each device, and the corresponding time duration of the whole experiment was 6 h. The frequency range during each data collection is 10 Hz–5 kHz. In EIS analysis for metal corrosion, the lowest frequency is essential due to the diffusion phenomenon that can be detected^7^. In our experiment, the selection of the lowest signal frequency is 10 Hz, which was a trade-off between the EIS analysis requirement and the testing efficiency. The selection of the highest frequency of 5 kHz was based on the result of the precision verification. In the precision verification that was presented earlier, the highest frequency was 6 kHz. The selection of 5 kHz can make sure that the proposed FDICS works in a typical frequency region. Table 5 in the “Appendix” section gives all the frequency points used in the EIS analysis. The value grows linearly per decade. All the impedance magnitude results from both of the devices were shown in Fig. 7a, we can see that the impedance magnitude decreases as testing time elapses (1.5 h, 3 h, etc.). The decrease of the magnitude over frequency corresponds to the fact that the corroded surface is capacitive in nature. From Fig. 7a, we can also see that the agreement between the magnitude results from FDICS and MFIA. Figure 7b shows the measured phase of the impedance of the sample. As frequency increases, the value was closer to zero degree. From Fig. 7b, it is noticeable the results from the FDICS, and the MFIA showed some deviation (below 200Hz) while the overall trend stayed the same.

**Figure 8:**
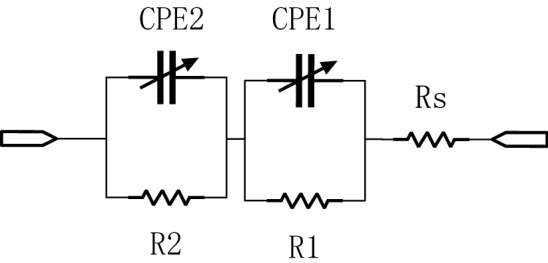
The equivalent circuit.

**Figure 9:**
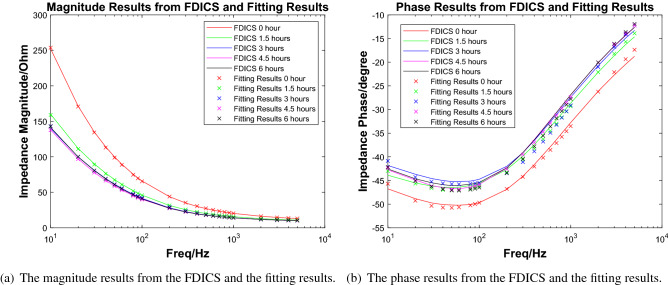
The impedance measurement from FDICS and the fitting result.

**Figure 10:**
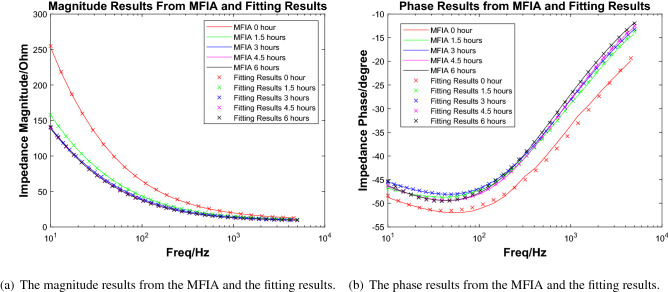
The impedance measurement from MFIA and the fitting result.

The next step is the EIS data fitting process, Fig. 8 shows the equivalent circuit(Voigt circuit) that we used to do the data fitting. The same circuit has been used in^19^ to do the data fitting, which is under a very similar scenario to us. For the EIS fitting software, we used EC-LAB (BioLogic Science Instruments, Seyssinet-Pariset, Auvergne Rhone Alpes) software to fit the data. The fitting methods were “Randomize and Simplex”. In this method, the software randomly selected parameters for fitting. The software searched for optimal initial values. Then the software does the simplex minimization, which was often used to minimize linear functions. Through the optimization of these two steps, we could obtain relatively accurate equivalent circuit parameters^27^. The fitting of each set of data has a total of 5000 iterations. The fitting was performed for the data we got from the FDICS and the MFIA. Figure 9a, b show the data from the FDICS and fitting result, while Fig. 10a, b show the data from the MFIA and the fitting result. Table 6 in the “Appendix” section shows the result of the data fitting, where we have listed the parameters of the equivalent circuit at the different time points of measurements.

## Discussion

The Voigt shown in Fig. 8 is commonly used as an equivalent circuit^28^. It has been rigorously derivated by Agarwal et al. that if the given impedance data is well fitted with the Voigt circuit, the data should satisfy the Kramers–Kronig relationships^29^. In Table 6 the fitting error was given in the form of Chi-Square result, which is a common way of presenting the fitting error in EIS analysis^30^. In advanced software such as the EC-LAB, the chi-square results can be generated simultaneously when the fitting data program is running. Typically, an acceptable fitting yields the chi-square result of value between $10^{-2}$ to $10^{-4}$^30–32^. As shown in the Table 6, chi-square results were given to demonstrate the goodness of the fitting in our experiment. And we can conclude that the impedance data from experiments satisfiy the Kramers–Kronig relationships.

In the circuit, Rs represents the resistance of the electrolyte solution^23^. The value of Rs is closely related to the factors such as the concentration of the solution and temperature. As the corrosion reaction progresses, the composition of the electrolyte solution in the corrosion cell evolves over time, and the conductivity of the solution changes; as shown in the Table 6, Rs will also vary as the reaction progresses. For the constant phase element (CPE), its impedance is given in the Eq. (6). In the equation, Q is the CPE value. CPE is an element that can model the behavior between a pure capacitor and a pure resistor. $\alpha$ is the exponential value that determines the CPE model’s characteristic and takes the value between 0 and 1. When $\alpha =0$, the CPE model is purely resistive. On the contrary, when $\alpha =1$, it is strictly capacitive. The constant phase element is related to the double-layer capacitance that represents the capacitance of the electrical double-layer that sticks to the metal sample surface. CPE is used instead of an ideal capacitor model because we want to obtain a more accurate fitting result and it is common to see that the CPE is treated as a replacement model for the capacitor since more degree of freedom is added into the EIS analysis^33^. The value of $\alpha$ for which the value usually lies between 0.5 and 1^33^.

6 $$Z=\frac{1}{Q(j\omega )^{\alpha }}$$

In the figure, whether it is the magnitude result or the phase result, we can see that a larger part of the lines corresponding to 3 h, 4.5 h and 6 h overlap. At these three points, the measured impedance result didn’t differ from each other as the data from 0 and 1.5 h. In this case, we can consider that the corrosion reaction has progressed to the limit. The impedance value will change very slowly, but we should note that in this case, different measurement results will also be caused by the instrument itself.

CPE2 and R2 in the equivalent circuit represent the corrosion layer^19^. Same as the discovery in^19^,in which the researchers explained the relationship between charge transfer resistor and the protective function of the corrosion layer, we also find a non-negligible charge transfer resistance, which is in the order of hundred Ohms.Grassini et al.^19^ explained the large value indicates that the corrosion layer is protective and the thicker corrosion layer cannot form. Other than noticing its value, we found an overall decreasing trend of the charge transfer resistance. The charge transfer resistance shows the difficulty of the charge to transfer between substances during the corrosion reaction on the surface of the metal. As shown in the Table 6, the value of Rt dropped as time prolong in the experiment. The study finds that the decrease is related to the chloride ion facilitate the corrosion on the carbon steel. $\hbox {FeCl}_{2} * \hbox {4H}_{2}\hbox {O}$ as a mid product for the reaction that accelerates the reaction of the iron^34^.

Table 4 shows the comparison of the proposed FDICS to the other 3 different devices. The commercial SP-200 workstation listed in the table is a benchtop machine with outstanding performance. Compared to the other two works in reported literature, the proposed FDICS shows the good capability to conduct EIS analysis on the metal corrosion sample.

**Table 4:** Comparison the proposed FDICS to other devices.

Device	SP-200 workstation	Arduino-based EIS system^19^	Impedance detection circuit^14^	This work
Accuracy	0.03%	$<5\%$ for magnitude $< 3$ for phase	3%	2.8% magnitude, 1.051 error in phase
Effective frequency range	$10\, \upmu \hbox { Hz}$ to 7 MHz	0.01 Hz–100 kHz	Up to 100 kHz	10 Hz–200 kHz (Resistor), 10 Hz–5 kHz (EIS)
Is it potentiostat?	Yes	Yes	No	Yes
Application	Multiple	Metal corrosion monitoring	Bio-molecules detection	Metal corrosion monitoring

## Conclusion

We have proposed an in-situ EIS analysis system that can perform in-situ EIS analysis of the corroded metal sample while decreasing in size and portability increases. Hardware and software development was introduced to show the functionality of the device. The design of the corrosion cell increases the portability of the whole setup. Experiments have been conducted to show the capability and accuracy of the proposed FDICS under a certain frequency range. Precision verification shows the accuracy of the device. Finally, an in-situ EIS analysis has been performed on a corroded metal sample. The EIS analysis data acquired by the proposed FDICS showed how the corrosion cell change over time. Also, the bahevior of the data can be explained based on reported literature.

## Figures and Tables

**Figure 11:**
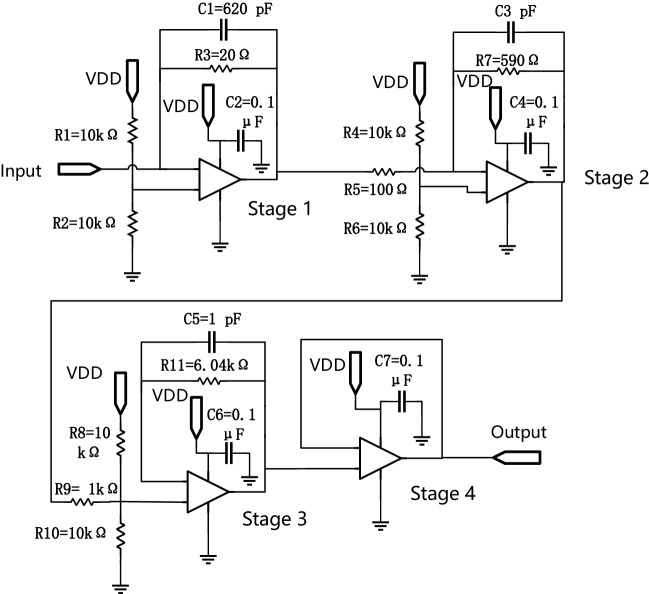
The TIA with driver used in the system.

**Figure 12:**
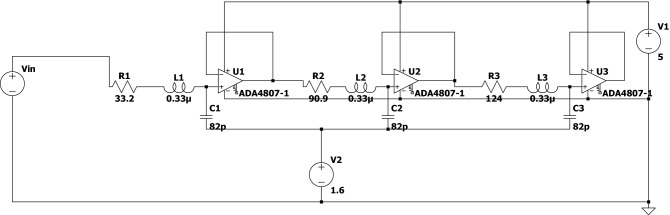
The low-pass filter schematic.

**Figure 13:**
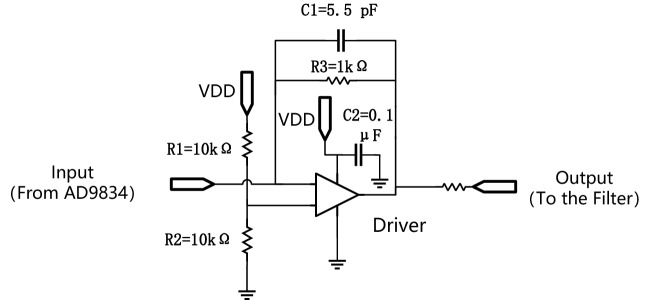
The driver schematic.

## Appendix

In the whole system, the most challenging part is the design of the analog-front-end. For the design schematic of the DDS, analog switch, and Arduino, the design is based on their official design published by their company. Here, we give the schematic of the custom-designed schematic. They are the trans-impedance amplifier (Fig. 11), the low-pass filter (Fig. 12) and the driver circuit (Fig. 13).

**Table 5:** The testing frequencies used in the EIS analysis.

Frequency/Hz	10	20	30	40	50	60	70	80	90	100	200	300
Frequency/Hz	400	500	600	700	800	900	1000	2000	3000	4000	5000

**Table 6:** The fitting result of EIS data from the FDICS and MFIA.

Time	Rs $\Omega$	R1 $\Omega$	CPE1: Q1 F*s⌃($\alpha-1)$	CPE1: $\alpha$1	R2 $\Omega$	CPE2: Q2 F*s⌃($\alpha-1)$	CPE2: $\alpha$2	Fitting Error: Chi-Square result
**Fitting result for the data from FDICS**								
0 h	10.13	249.4	0.7063e−3	0.5938	578.1	0.2349e−3	0.7583	4.482e−3
1.5 h	9.12	114.4	0.6584e−3	0.6421	482.1	0.4712e−3	0.7307	4.394e−3
3 h	8.98	24.7	0.4821e−3	0.7351	339.4	0.3043e−3	0.7528	20.240e−3
4.5 h	8.82	184.2	0.4095e−3	0.6707	249.9	0.5343e−3	0.9096	1.680e−3
6 h	9.04	100.9	0.4529e−3	0.6800	310.2	0.3836e−3	0.8282	11.04e−3
**Fitting result for the data from MFIA**								
0 h	9.30	257.7	0.4159e−3	0.6219	702.1	0.2086e−3	0.8404	24.610e−3
1.5 h	8.64	137.6	3.3530e−3	0.6092	663.8	0.3694e−3	0.6609	2.433e−3
3 h	8.18	12.03	0.7778e−3	0.8861	787.0	0.4966e−3	0.6468	71.790e−6
4.5 h	7.99	17.19	1.1770e−3	0.7917	665.0	0.4508e−3	0.6741	0.450e−3
6 h	8.31	161.9	8.6620e−3	0.6197	444.4	0.3787e−3	0.6897	2.054e−3
